# High throughput mRNA sequencing reveals potential therapeutic targets of Si-Ni-San in the pons for a stress-induced depression model

**DOI:** 10.3389/fphar.2024.1383624

**Published:** 2024-07-29

**Authors:** Junling Li, Yan Zhang, Te Li, Binbin Nie, Fang Qi, Qijun Chen, Tianxing Chen, Yuhang Liu, Gaifen Li, Yubo Li

**Affiliations:** ^1^ School of Traditional Chinese Medicine, Capital Medical University, Beijing, China; ^2^ Department of Traditional Chinese Medicine, Beijing Friendship Hospital, Capital Medical University, Beijing, China; ^3^ Key Laboratory of Nuclear Analytical Techniques, Institute of High Energy Physics, Chinese Academy of Sciences, Beijing, China; ^4^ Institute of Basic Theory for Traditional Chinese Medicine, China Academy of Chinese Medical Sciences, Beijing, China

**Keywords:** CUMS, Si-Ni-San, pons, antidepressant mechanism, RNA-seq, fMRI

## Abstract

**Background:**

An accumulating body of research indicates that the pons is related to the occurrence of depression. Si-Ni-San (SNS) is a well-known Chinese herbal formula that is used to treat depression. Chinese herbal formulae have multiple therapeutic characteristics. Although it has been proven that SNS can exert antidepressant effects by improving changes in the limbic system, it is currently unclear whether SNS has therapeutic targets in the pons. This study aimed to explore the therapeutic targets of SNS in the pons for depression treatment.

**Materials and methods:**

Two experiments were conducted. In Experiment 1, 32 rats were divided into four groups: (1) a Control (C) group that received distilled water as a vehicle; (2) a Model (M) group that received the chronic unpredictable mild stress (CUMS) procedure and was administered distilled water; (3) a Stress + SNS (MS) group that received the CUMS procedure and was administered SNS dissolved in distilled water; and (4) a Stress + Fluoxetine (MF) group that received the CUMS procedure and was administered fluoxetine dissolved in distilled water. The open field test (OFT), the sucrose preference test (SPT), and the novel object recognition test (NOR) were performed to test the antidepressant effects of SNS. High-throughput mRNA sequencing (RNA-seq) was used to explore possible gene targets of SNS in the pons, and quantitative real-time PCR was performed to verify the results. High-performance liquid chromatography was used to detect neurotransmitters. Finally, correlation analyses were conducted between behaviors, genes expression, and neurotransmitters. In Experiment 2, 18 rats were divided into the same three groups as in Experiment 1: (1) C, (2) M, and (3) MS. fMRI was used to confirm whether SNS altered the pons in a rat model of depression.

**Results:**

SNS significantly improved sucrose preference in the SPT and T_N_-T_O_ in the NOR compared to the M group (*P* < 0.05). RNA-seq filtered 49 differentially expressed genes(DEGs) that SNS could reverse in the pons of the CUMS depression model. Real-time PCR detected six genes, including Complexin2 (Cplx2), Serpinf1, Neuregulin1 (Nrg1), Annexin A1 (Anxa1), β-arrestin 1 (Arrb1) and presenilin 1 (Psen1). SNS significantly reversed changes in the expression of Anxa1, Nrg1, and Psen1 caused by CUMS (*P* < 0.05), which is consistent with the DEGs results. Additionally, SNS significantly reversed norepinephrine (NE) changes in the pons. There were 18 noteworthy correlations between behavior, genes, and neurotransmitters (*P* < 0.05). fMRI showed that SNS can decrease the amplitude of low-frequency fluctuations (ALFF) in the pons of living depressed rats.

**Conclusion:**

The pons is an important target brain region for SNS to exert its antidepressant effects. SNS may improve pontine NE levels by regulating the Anxa1, Nrg1, and Psen1 genes, thereby exerting antidepressant effects and improving cognitive function.

## 1 Background

Major depressive disorder (MDD) is characterized by persistent low mood and loss of interest, often accompanied by cognitive dysfunction and various physical symptoms (Abdoli et al., 2022). In 2008, the World Health Organization (WHO) ranked it as the third-highest disease burden worldwide and projected that it would rank first by 2030 (WHO, 2008). Individuals with MDD are at high risk of developing comorbid anxiety and substance use disorders, which further increase their risk of suicide. Psychotherapy and pharmacological treatments have remained the first-line treatments along with selective serotonin reuptake inhibitors, which are usually the first choice of medication (Bains and Abdijadid, 2023). The pathogenesis of depression is relatively complex, and exploration of its mechanisms involves studying several brain regions associated with the disease. Of these regions, the hippocampus, the amygdala, and the anterior cingulate cortex have been the most extensively studied; each of these are part of the limbic system. Antidepressant treatments targeting these brain regions have shown beneficial effects (Pandya et al., 2012). However, according to clinical surveys, more than 50% of patients do not respond effectively to modern first-line antidepressant medications, and over 70% of patients are unable to achieve full recovery (Hou et al., 2016). Hence, there is still a need for an in-depth exploration of the specific pathogenesis of depression and its prevention and treatment strategies (Malhi and Mann, 2018).

Although most previous studies have focused on the limbic areas when exploring the pathophysiology of depression and its antidepressant efficacy, growing evidence suggests that many brain regions, in addition to the limbic system, are dysfunctional in animal and human models of depression (Zhang et al., 2018). The pons are located at the bottom of the skull. It is one of the most basal structures in the brain and is part of the brainstem. The latest clinical research shows that the pons are related to emotional responses and the processing of emotional information (Wong et al., 2020). The strength of the connection between the pons and the amygdala is positively correlated with the severity of depressive symptoms (Wong et al., 2022). In addition, our previous study showed that pontine nerve signaling activity is significantly correlated with learning and cognitive function (Li et al., 2018). Therefore, the pons are involved in emotional processing in depression and is a noteworthy brain region for studying depression. However, all previous studies have only suggested a close relationship between the pons and depression based on neuroimaging findings, few studies have investigated the molecular biological mechanisms underlying the involvement of the pons in depression. Hence, it would be worthwhile to conduct an in-depth study of the pontine region.

Traditional Chinese Medicine (TCM) is a form of complementary and alternative medicine that has shown satisfactory effects against many modern diseases. Si-Ni-San (SNS) is a TCM herb formula that was derived from the classics of TCM Shang Han Lun (Treatise on Febrile Diseases). It constitutes of Bupleuri radix (*Bupleurum chinense* DC, CH), Paeoniae radix alba (*Paeonia lactiflora* Pall., BS), Aurantii fructus immaturus (*Citrus trifoliata* L, ZS), and Glycyrrhizae radix et rhizoma praeparata cum melle (*Glycyrrhiza uralensis* Fisch., GC) in a ratio of 1:1:1:1. In TMC clinics, SNS have shown promising effects on MDD (Guan and Qu, 2023; Qiao et al., 2023). Although it has been proven that SNS can exert antidepressant effects by improving changes in the limbic system -- such as the hippocampus, the hypothalamus, and the amygdala (Yan et al., 2005; Li et al., 2018; Deng et al., 2022) -- it is currently unclear whether SNS has therapeutic targets in the pons.

High-throughput RNA sequencing (RNA-seq) is a robust transcriptional screening technology that can identify differentially expressed genes (DEGs) by comparing different conditions such as normal and diseased states (Finotello and Di Camillo, 2015; Luo et al., 2020). It is a powerful tool for investigating the pathogenesis of diseases and for developing new drugs for treatment. In recent decades, high-throughput RNA-seq has been increasingly used to explore the pathogenesis of depression and the mechanisms of action of antidepressants (Xiong et al., 2021; Li et al., 2022). Resting-state functional magnetic resonance imaging (r-fMRI), which measures intrinsic or spontaneous neural activity *in vivo*, has become increasingly popular for studying MDD pathophysiology. Amplitude of low-frequency fluctuations (ALFF) is an r-fMRI analysis method that can detect regional neural activity across the brain (Yang et al., 2007). Hence, it provides a method for exploring the possible pathology of brain regions involved in depression as well as the brain region targets for antidepressants. Chronic unpredictable mild stress (CUMS) is currently the most commonly used, reliable, and effective rodent model for studying the neurobiological basis of depression (Antoniuk et al., 2019). In our previous study, we used this method to establish a stable depression rat model (Li et al., 2018). Hence, in the present study, based on this model, we conducted RNA-Seq to explore the potential therapeutic targets of SNS in the pons and used fMRI to further confirm whether SNS can improve pontine functional activity in living rats with depression. This study will fill the gaps in the potential molecular biological basis relating the pons to depression, as well as the target of the anti-depression effect of SNS in the pons.

## 2 Methods

### 2.1 Animals

Two experiments were conducted. Six-week-old male Sprague-Dawley (SD) rats provided by Beijing Weitong Lihua Experimental Animal Technology Co., Ltd. were used in the two experiments. For Experiment 1, 32 rats were housed in the animal center of the Capital Medical University. The animals were housed under a controlled light/dark cycle (12:12 h, lights on at 06:00) and temperature/humidity (22°C, 30%–40%). For Experiment 2, 18 rats were housed in the animal center of Beijing University of Chinese Medicine under the same conditions as those used in Experiment 1. All animal experiments were reviewed and approved by the local committee (Ethics approval number for Experiment 1: AEEI-2018-009; Ethics approval number for Experiment 2: 2013BZHYLL1001B).

### 2.2 Grouping

All animals were adaptively fed for 2 weeks before being randomized into different groups.

For Experiment 1, 32 rats were divided into four groups: (1) a control (C) group that was administered distilled water as a vehicle; (2) a model (M) group that received the CUMS procedure and distilled water; (3) a Stress + SNS (MS) group that received the CUMS procedure and SNS dissolved in distilled water; and (4) a Stress + Fluoxetine (MF) group that received the CUMS procedure and fluoxetine dissolved in distilled water. In Experiment 2, 18 rats were divided into the same three groups from Experiment 1: (1) C group, (2) M group, and (3) MS group.

For both Experiments 1 and 2, the rats received either the drug or vehicle daily through oral gavage immediately prior to the stress procedure. The rats in Group C were housed in groups of four, whereas the other rats were housed in isolation.

### 2.3 Drug administration

The rats in each group were administered 1 mL/100 g of liquid medicine or distilled water via gavage. The rats in the MF group in Experiment 2 were treated with fluoxetine (2 mg/kg/day, Patheon France, packaged by Lilly Suzhou Pharmaceutical Co., Ltd., Suzhou, China) (Zhou et al., 2021) and the rats in the C and M groups received distilled water by gavage. The four Chinese medicinal herbs that make up SNS were dispensed granules manufactured by Jiangyin Tianjiang Pharmaceutical Co., Ltd. The production batch number and its ratio to the raw materials were as follows: Bupleuri radix (batch 19012491 at a 1:6 ratio); Paeoniae radix alba (batch 19010151 at a 1:10 ratio); Aurantii fructus immaturus (batch 18121851 at a 1:12 ratio); and Glycyrrhizae radix et rhizoma praeparata cum melle (batch 18111001 at a 1:6 ratio). The granules were dissolved in distilled water to prepare an SNS suspension containing crude drug at a concentration of 0.25 g/mL. The dose of SNS used in the two experiments was 2.5 g/kg/day, as described previously (Li et al., 2015).

### 2.4 CUMS procedure

In both experiments, 8-week CUMS was conducted on all rats except for the C group. In the first experiment, CUMS was performed as described previously (Li et al., 2018). In the second experiment, CUMS was performed with minor changes according to the laboratory conditions. Briefly, there were ten stimulations applied to the experimental rats as follows: food deprivation for 12 h, water deprivation for 12 h, wet housing overnight, forced swimming at 4°C for 5 min, restraint for 2.5 h, overnight illumination, noise for 12 h, stroboscopic stimulation for 12 h, crowding for 12 h, and tail clamping for 1.5 min. The rats were exposed to a random selection of two stressors from the above stimulations per day with no repetition of the same type on consecutive days, which guaranteed that the animals faced unpredictable stimulation.

### 2.5 High performance liquid chromatography (HPLC) for SNS quality control

To prepare the SNS sample, SNS granules equivalent to 0.6 g of the raw material were dissolved in 37.5 mL methanol with ultrasound for 10 min, and a 0.016 g/mL solution was obtained by filtration. In order to prepare the reference solution, Paeoniflorin, Liquiritin, Naringin, Saikosaponin A, Saikosaponin D, and Glycyrrhetinic acid were accurately weighed, and methanol was added to make a solution containing 1.81 mg per milliliter.

**FIGURE 1:**
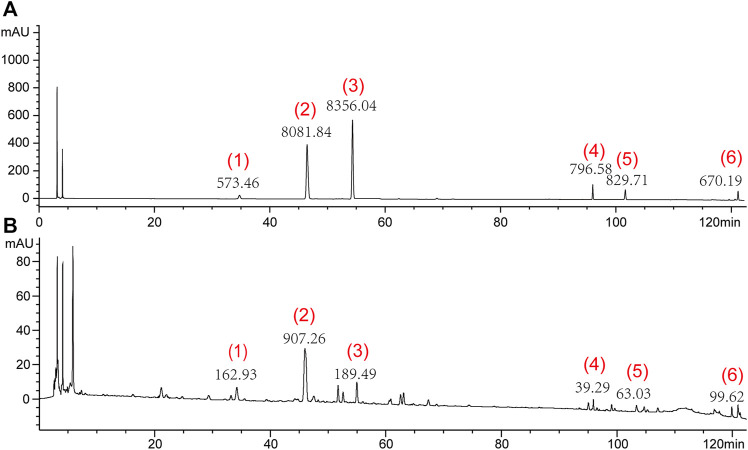
Representative high-performance liquid chromatograms of mixture of standard compounds **(A)** and SNS **(B)**. (1) Paeoniflorin, (2) Liquiritin, (3) Naringin, (4) Saikosaponin A, (5) Saikosaponin D, and (6) Glycyrrhetinic acid.

The HPLC analysis was performed using an Agilent 1100 liquid chromatography system (Agilent 1200, Agilent, United States). A 10 μL sample was injected into a TC-C18 column (4.6 mm× 250 mm, 5 μm) and eluted with a mobile phase gradient of acetonitrile (A) and 0.05% phosphoric acid (B) at a flow rate of 1.0 mL/min. The gradient elution program used a method described previously (Li and Huang, 2005), as follows: 0–10 min (10% A), 10–30 min (10%–12% A), 30–35 min (12%–18% A), 35–40 min (18% A), 40–42 min (18%–20% A), 42–62 min (20%–28% A), 62–70 min (28% A), 70–75 min (28%–30% A), 75–80 min (30%–33% A), 80–85 min (33% A), 85–95 min (33%–50% solvent A), 95–105 min (50% A), and 105–122 min (50%–90% A). The analytes were detected at a wavelength of 210 nm. Under these conditions, the six main components were well separated; the peak heights and areas of each component are shown in Figure 1.

### 2.6 Experiment 1

#### 2.6.1 Open field test (OFT)

The animals were placed individually in the center of an open-field box (100 cm × 100 cm), and their spontaneous motor activity was recorded for 3 min. Rearings were manually counted during recording. An animal behavior analysis system was used to analyze the total distance moved over a 3 min period.

#### 2.6.2 Sucrose preference test (SPT)

The SPT was performed as previously described (Li et al., 2018). Briefly, after adaptive training and deprivation of water and food for 24 h, the rats were housed in individual cages and provided free access to two bottles of water and sucrose. After 4 h, sucrose solution and water consumption were recorded. Sucrose preference was calculated as follows: sucrose preference = sucrose consumption (g) × 100%/ [sucrose consumption (g) + water consumption (g)].

#### 2.6.3 Novel object recognition test (NOR)

NOR was performed as previously described (Li et al., 2018). 24 h after the two habituation sessions, the rats were returned to the testing apparatus for the experimental session, which consisted of two trials. During the first trial (T1), the rats were exposed to two identical objects, either Old1(O1) or Old2(O2). After 60 min, in the second trial (T2), the rats were exposed to two different objects, O1 or O2, and a new object, New(N). The time spent exploring the two objects at T2 was recorded. Object recognition was defined using variables T_N_-T_O_ and T_N_-T_O_/T_N_+T_O_. After completion of all behavioral experiments, the rats were sacrificed and their brains were immediately removed. The pons tissues were extracted on ice and quickly frozen in liquid nitrogen and stored at −80°C.

#### 2.6.4 Transcriptome sequencing and analyses

Three animals were randomly selected from each group (C, M, and MS) for RNA-Seq analysis. Sequencing and transcriptome analyses were performed by Sangon Biotech Co., Ltd. (Shanghai, China). Briefly, total RNA was extracted using TRIzol reagent (Cat# B511311, Shanghai Sangon Biotech) according to the manufacturer’s instructions. The quantity and quality of the RNA samples were determined using spectrophotometry and gel electrophoresis, respectively. Hieff NGS™ MaxUp Dual-mode mRNA Library Prep Kit for Illumina^®^ (YEASEN Biotech, Shanghai) was used to prepare libraries following the manufacturer’s instructions, and purified libraries were quantified using a Qubit2.0 DNA detection kit (Life Technologies, United States). Sequencing was performed on the Illumina NovaSeq 6000 platform using a paired-end readout module.

After sequencing, raw data were assessed using FastQC (version 0.11.2) and low-quality reads were removed using Trimmomatic (version 0.36) to ensure data accuracy and effectiveness. RNA-seq reads were mapped to the *Rattus norvegicus* reference genome (Rnor_6.0) using HASAT2 (version 2.1.0) and the location of the short sequence of the sample in the reference genome sequence was recorded using RSeQC (version 2.6.1). The homogeneity of the distribution and genome structure was checked using QualiMap (version 2.2.1). Statistical analysis of gene coverage ratios was performed using BEDTools (version 2.26.0). DEGseq (version1.26.0) was used to determine differential gene expression, with significance determined by a *p*-value < 0.05, and an absolute value of a log 2-foldchange >2. The Q-values were adjusted using the Benjamini-Hochberg method (Benjamini and Hochberg, 1995). TopGO (version 2.24.0) was used for the GO enrichment analysis. Based on these results, the top10 GO-terms and related significant DEGs were selected to construct a significant enrichment function-gene interaction network using the igraph package of R (version 1.0.1).

#### 2.6.5 Validation of differentially expressed mRNAs from the sequencing profile by real-time PCR

Quantitative real-time PCR was performed to verify the RNA-seq results using GAPDH as an internal control. Relative expression of mRNAs was determined using the 2^-△△CT^ method. The following six genes were analyzed: Complexin2 (Cplx2), Serpinf1, Neuregulin1 (Nrg1), Annexin A1 (Anxa1), β-arrestin 1 (Arrb1), and presenilin 1 (Psen1). PCR primer sequences are shown in Table 1. Pons tissues from the C, M, MS, and MF groups were analyzed (24 samples).

**TABLE 1:** Primer sequences.

Gene	Primers sequences	PCR product length (bp)
GAPDH	F: CAA​GTT​CAA​CGG​CAC​AGT​CAA	140
	R: CGC​CAG​TAG​ACT​CCA​CGA​CA	
Cplx2	F: CGAGGGAAGCCTGACCAG	105
	R: GGC​AGA​TAT​TTG​AGC​ACC​GT	
Serpinf1	F: ACT​GGC​AAC​CCT​CGC​ATA​G	202
	R: TGT​CCT​CGT​CCA​AGT​GAA​AA	
Nrg1	F: AAA​ACT​TTC​TGT​GTG​AAT​GGG​G	174
	R: GTA​GAG​TTC​CTC​CGC​TTC​CAT	
Anxa1	F: CCC​TAC​CCT​TCC​TTC​AAT​CC	221
	R: ATA​GCC​AAA​ACA​ACC​TCC​TCC	
Arrb1	F: TGG​AGA​ACC​CAT​CAG​CGT​C	209
	R: TTG​CCA​GGA​AGG​GAG​TCA​G	
Psen1	F: CAT​TCA​CAG​AAG​ACA​CCG​AGA​C	141
	R: CAT​GGA​TGA​CCT​TGT​AGC​ACC	

#### 2.6.6 Neurotransmitter detection

After weighing, the tissues were homogenized at 4°C with 0.4 M/L perchloric acid in a 2 mL Eppendorf tube. The homogenate was centrifuged at 4°C for 20 min at 12,000 rpm, after which 90 uL of the supernatant was absorbed and 45 uL liquid containing 0.02 M/L potassium citrate; 0.3 M/L dipotassium hydrogen phosphate and 0.002 M/L EDTA·2Na was then added. The mixture was centrifuged at 4°C for 20 min at 12,000 rpm. The supernatant was collected and filtered through a 0.22 µm filter membrane before injection into the HPLC system. A reverse-phase column was used for norepinephrine (NE), dopamine (DA), 3, 4-dihydroxyphenylaceticacid (DOPAC), 5-hydroxytryptamine (5-HT), and 5-hydroxyindole acetic acid (5-HIAA) analysis. The mobile phase consisting of methanol (10%), citric acid monohydrate (63.5 mmol/L), trisodium citrate dehydrate (60.9 mmol/L), EDTA·2Na·2H2O (0.1 mmol/L), and sodium octaalkyl sulfonate (0.5 mmol/L), pH 4.3 was passed through the ODS separation column (150 mm× 3.9 mm) at a constant flow rate (0.5 mL/min). Neurotransmitter levels were expressed as ng/mg of wet tissue.

#### 2.6.7 Statistical analysis

SPSS 22.0 software (SPSS v.22.0; SPSS Inc., Chicago, IL, United States) was used to analyze the data. The Shapiro-Wilk test was used to examine data normality. One-way analysis of variance (ANOVA) was used to compare all groups if the data fit a normal distribution.To compare the two selected groups, an LSD *post hoc* test was used for data with homogeneity of variance, and Tamhane’s T2 test was used for those with heterogeneity of variance. If the data were not normally distributed, a nonparametric test with K independent samples was used. Pearson (data fit a normal distribution) or Spearman (data did not fit a normal distribution) correlation analyses were used to compare the indices of the behavioral tests, real-time PCR, and neurotransmitters. All data are expressed as the means ± standard deviation (SD). Statistical significance was set at *P* < 0.05.

### 2.7 Experiment 2

#### 2.7.1 fMRI acquisition

The procedure and relevant instrument parameters for the fMRI acquisition were the same as those used in a previous study (Li et al., 2018). Briefly, rats were anesthetized with isoflurane/O2. During the fMRI scanning process, the isoflurane concentration was decreased to 0.20%–0.25% to maintain a respiration rate of 60–85/min. A 7.0 T /16US MRI scanner was used to acquire T2-weighted data and resting-state functional images of the rat brain. After acquisition, all animals were decapitated under anesthesia.

#### 2.7.2 fMRI data analysis

Statistical parametric mapping (SPM12) and Resting-State fMRI Data Analysis Toolkit (REST) software were used to preprocess and statistically analyze the fMRI data. ALFF measurements were analyzed and compared among the C, M, and MS groups. The analytical method used was based on our previous study (Li et al., 2018).

## 3 Results

### 3.1 Behavioral experiments indicate that SNS improved depression related symptoms

**FIGURE 2:**
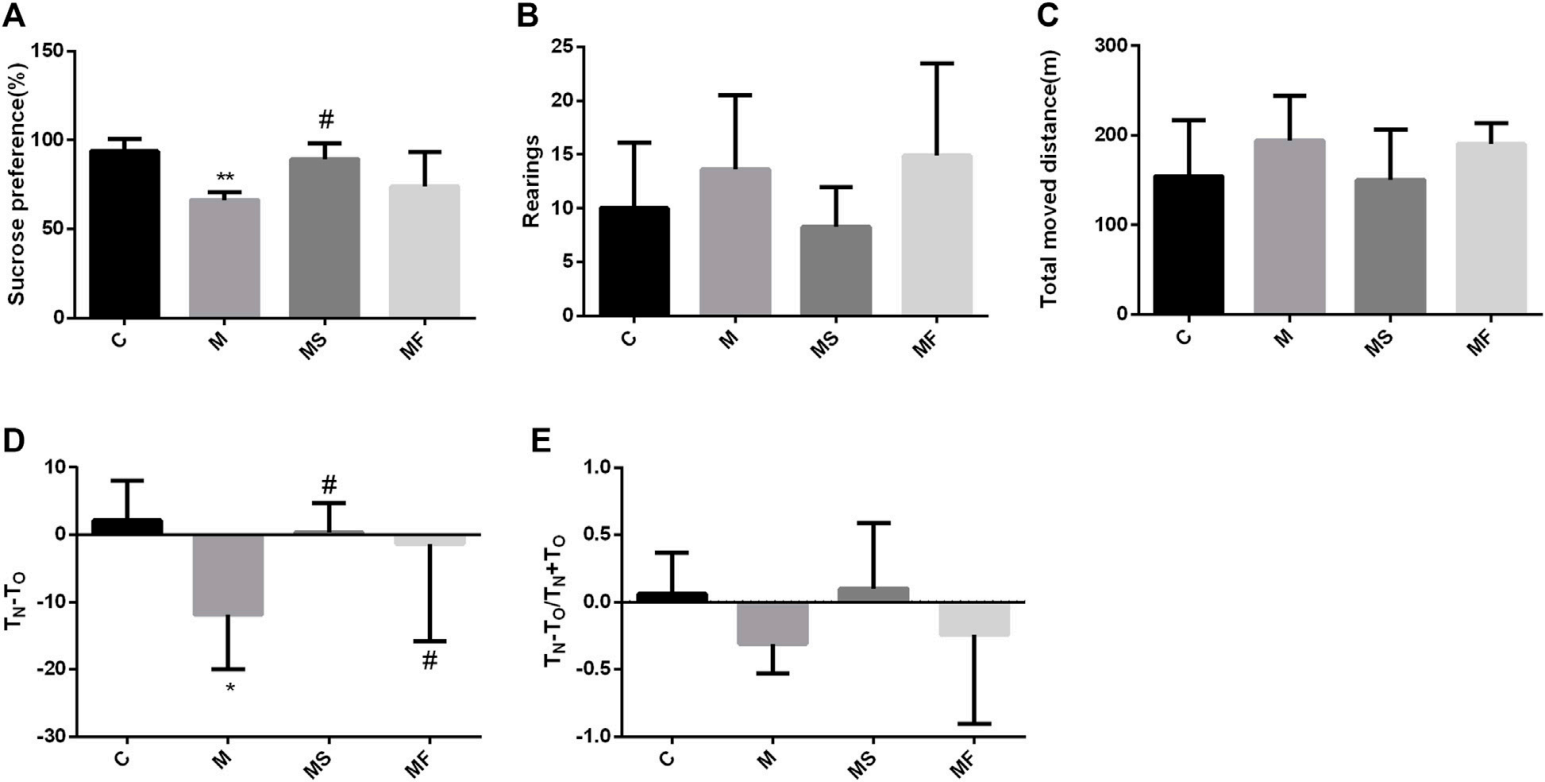
Effects of SNS on behavioral experiments. **(A)** Result of SPT. **(B)** Rearings in the OFT. **(C)** Total moved distance in the OFT. **(D)** T_N_-T_O_ in the NOR. **(E)** T_N_-T_O_/T_N_+T_O_ in the NOR. *P < 0.05 compared with C group. ^#^ *P* < 0.05 compared with M group.

Sucrose preference was significantly lower in group M than in group C. SNS significantly improved sucrose preference compared to that in group M (Figure 2A). In the OFT, the rats in each group exhibited no significant differences (Figures 2B, C). In the NOR, T_N_-T_O_ was significantly decreased in the M group compared to the C group, while SNS significantly improved it (Figure 2D). T_N_-T_O_/T_N_+T_O_ group showed no significant difference (Figure 2E).

### 3.2 Results of genome sequencing

#### 3.2.1 SNS reverse 49 pontine gene changes in the depression model

**FIGURE 3:**
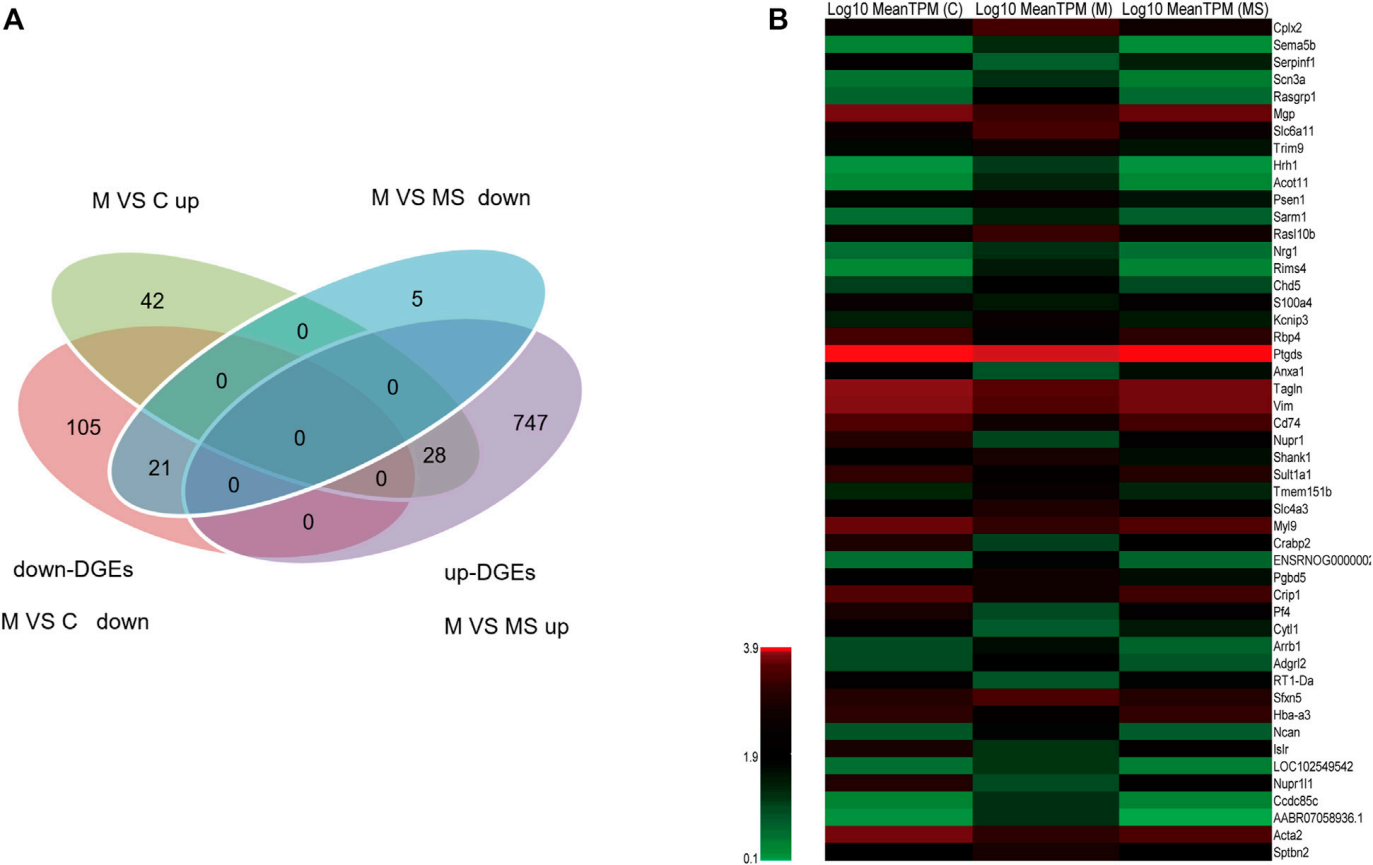
Identification of DEGs reversed by SNS in the pons. **(A)** The intersection gene of DEGs (M vs. C and M vs. MS, down- and upregulated, respectively). **(B)** The expression level of the 49 filtered DEGs in C, M and MS groups.

To further understand the multifaceted mechanisms of the SNS in depression, we performed RNA-seq to obtain the transcriptomes of C, M, and MS samples. As Figure 3A shows, 21 down-DEGs were identified at the intersection of 126 down-regulated mRNAs (M vs. C) and 26 down-regulated mRNAs (M vs. MS). Additionally, 28 up-DEGs were identified at the intersection of 70 upregulated mRNAs (M vs. C) and 775 upregulated mRNAs (M vs. MS). Hence, we filtered 49 DEGs that SNS could reverse in the pons of the depression model. The expression levels of 49 DEGs in each group are shown in Figure 3B.

#### 3.2.2 19 GO-terms were significantly enriched by the 49 DEGs and 14 genes related to the top 10 enriched GO-terms

**FIGURE 4:**
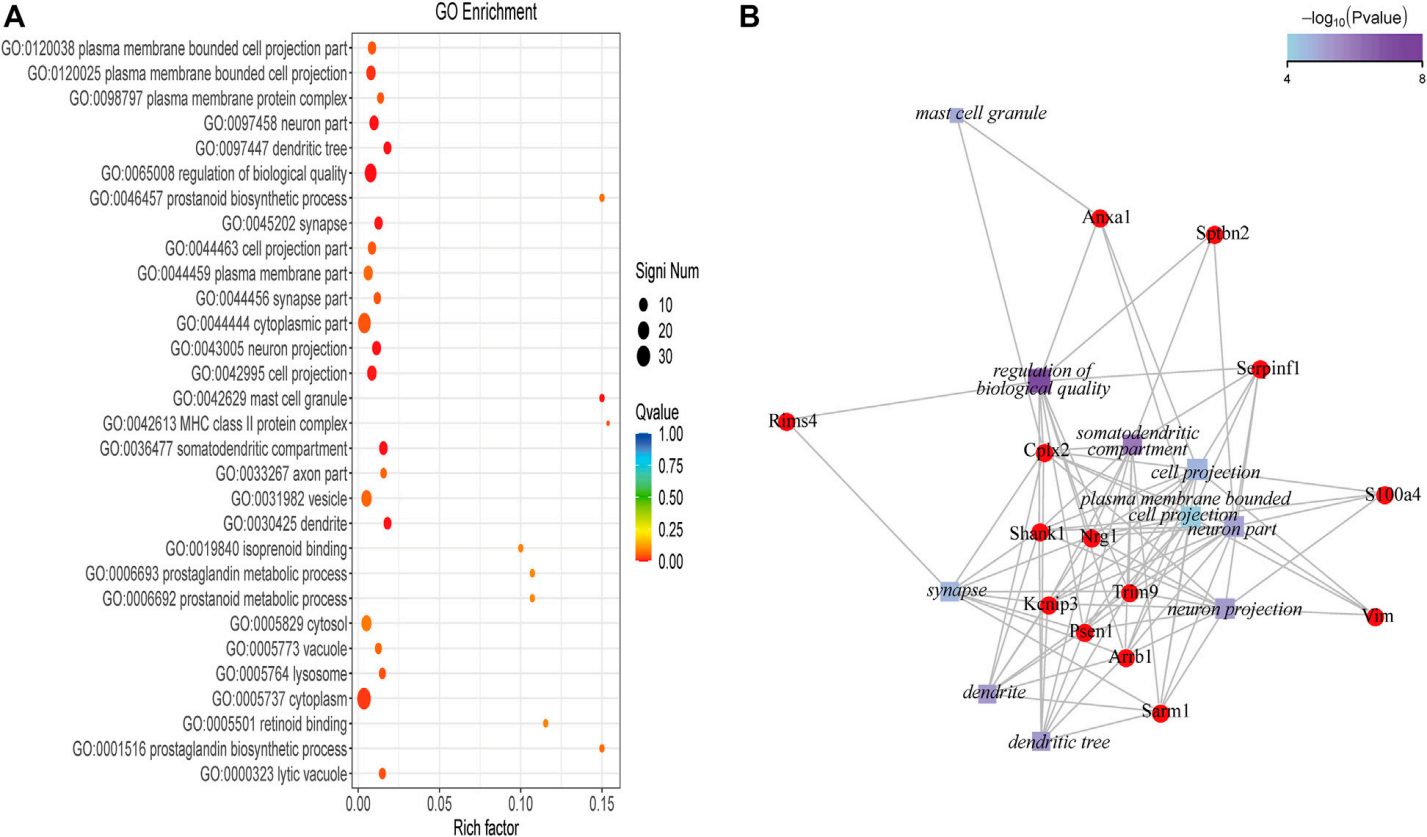
**(A)** The top 30 GO-terms with highest enrichment factor. **(B)** Network of the top 10 functions and the 14 related DEGs.

To further understand the DEGs related to the treatment of depression using SNS, GO enrichment analysis was performed on the 49 DEGs using ClusterProfiler. Figure 4A displays the top 30 GO terms with the highest enrichment factor. Of the GO terms, 19 were significantly enriched in the 49 DEGs, with one term belonging to the BP category and 18 terms belonging to the CC category, all with a q-value of <0.05. The top 10 enriched GO-terms were ranked as follows: regulation of biological quality (GO:0065008), somatodendritic compartment (GO:0036477), dendrite (GO:0030425), dendritic tree (GO:0097447), neuron projection (GO:0043005), neuron part (GO:0097458), mast cell granule (GO:0042629), cell projection (GO:0042995), synapse (GO:0045202), and plasma membrane bounded cell projection (GO:0120025). The top10 GO-terms and related significant DEGs were selected to construct a significant enrichment function-gene interaction network. The functional gene network consisted of 10 GO-terms nodes, 14 gene nodes, and 94 interactions, as shown in Figure 4B. Specific information of the 14 genes in the functional gene network is provided in Supplementary Table S1.

### 3.3 Real-time PCR further verified that SNS can improve Anxa1, Nrg1, and Psen1 changes in the pons

**FIGURE 5:**
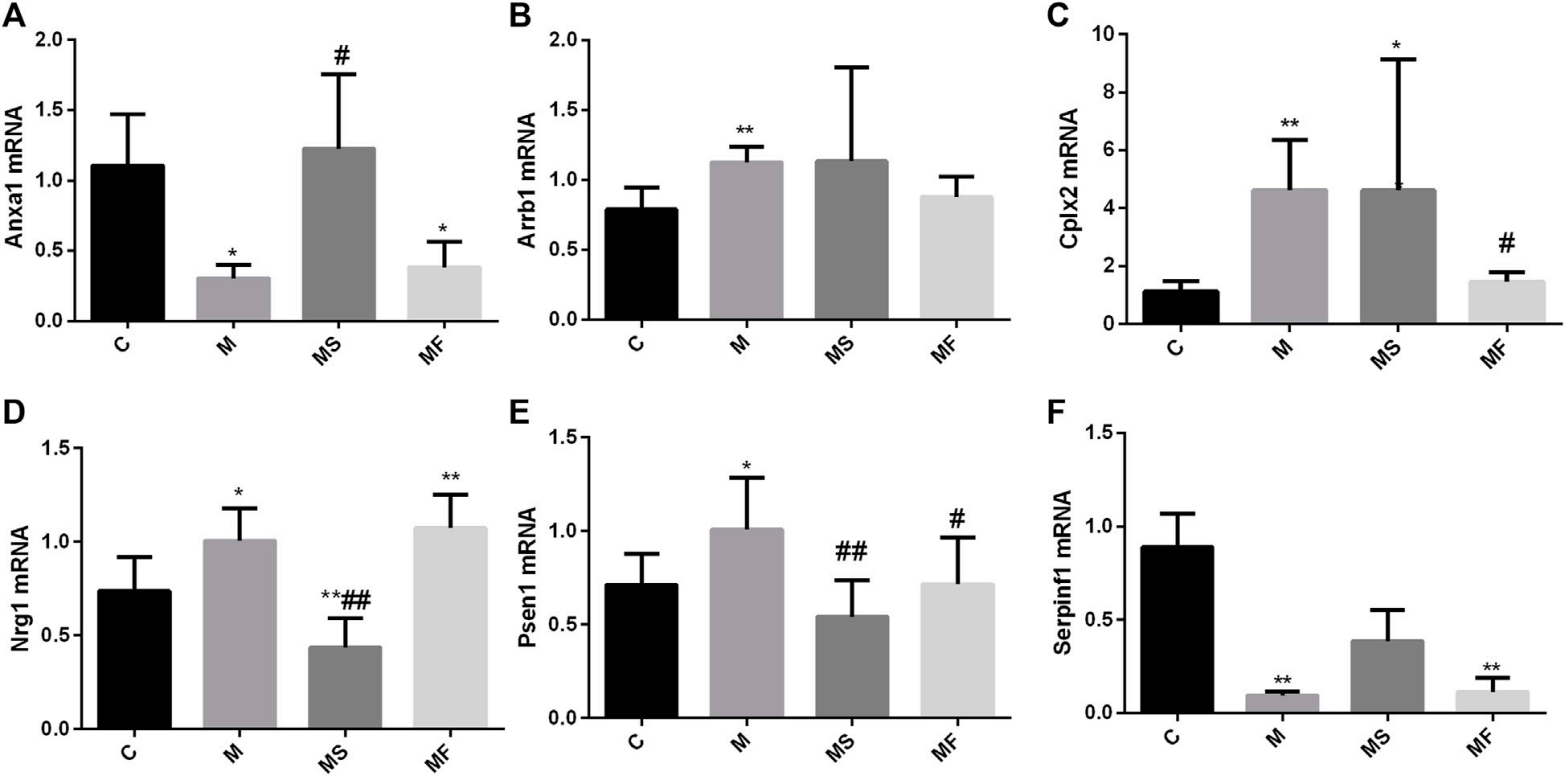
Effects of SNS on the genes in pons. **(A)** Anxa1, **(B)** Arrb1, **(C)** Cplx2, **(D)** Nrg1, **(E)** Psen1, **(F)** Serpinf1. *P < 0.05 compared with C group. **P < 0.01 compared with C group. ^#^ *P* < 0.05 compared with M group. ^##^ *P* < 0.01 compared with M group.

As shown in Figure 5, Arrb1, Cplx2, Nrg1, and Psen1 were overexpressed in the M group compared to those in the C group. Anxa1 and Serpinf1 showed low expression levels in the M group, as determined using real-time PCR. SNS significantly reversed the expression of Anxa1, Nrg1, and Psen1, but did not significantly affect the expression of Arrb1and Cplx2. Serpinf1 expression in the MS group showed an increasing trend, but this was not statistically significant. Fluoxetine significantly reversed the expression of Cplx2 and Psen1.

### 3.4 Neurotransmitter detection indicated that SNS reversed the pontine NE change

**FIGURE 6:**
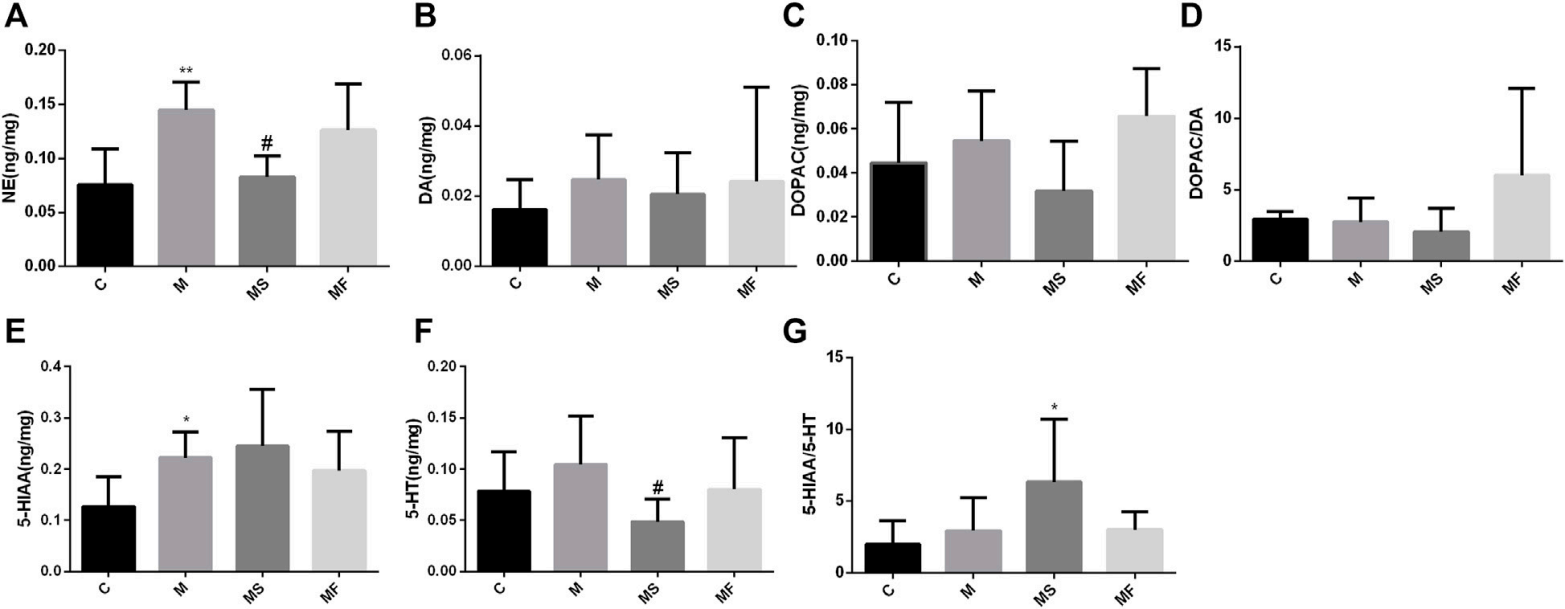
Effects of SNS on the neurotransmitters in pons. **(A)** NE, **(B)** DA, **(C)** DOPAC, **(D)** DOPAC/DA, **(E)** 5-HIAA, **(F)** 5-HT, **(G)** 5-HIAA/5-HT. **P < 0.05 compared with C group. **P < 0.01 compared with C group. ^#^ *P* < 0.05 compared with M group. ^##^ *P* < 0.01 compared with M group.

As shown in Figure 6, rats in the M group displayed a marked increase in the NE and 5-HIAA levels of the pons compared to those in the C group. SNS significantly reversed the NE change, but had no impact on 5-HIAA.

### 3.5 Correlation analysis showed that Anxa1, Nrg1, and Psen1 were significantly correlated with pontine NE and depression related symptoms

**FIGURE 7:**
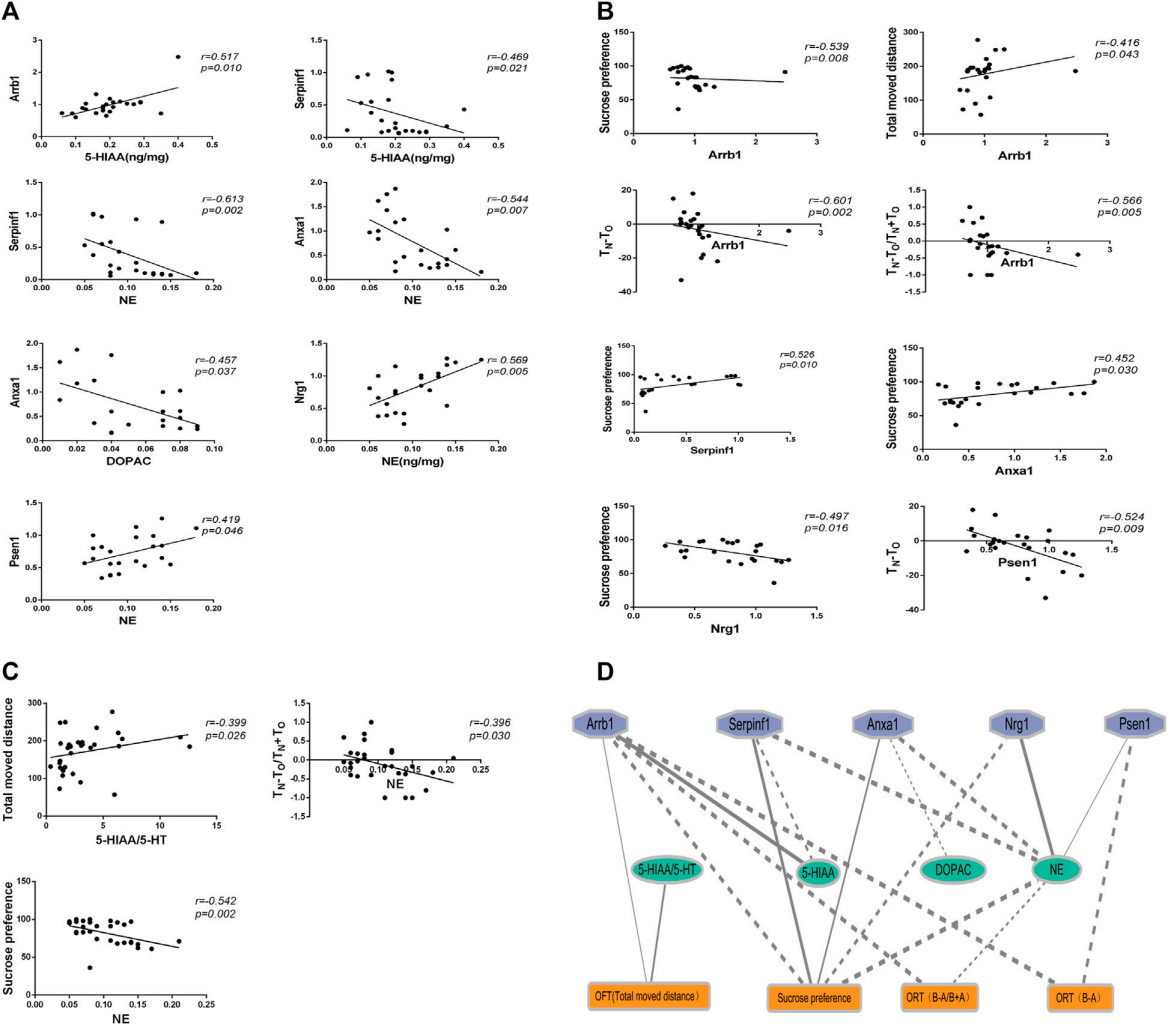
The correlation analysis between behaviors, genes expression, and neurotransmitters. **(A)** Correlation analysis between genes expression and neurotransmitters. **(B)** Correlation analysis between behaviors and genes expression. **(C)** Correlation analysis between behaviors and neurotransmitters. **(D)** Ethology-neurotransmitter-gene relationship map. Solid line indicates positive correlation, while dotted line indicates negative correlation. The smaller the *P* value, the thicker the line.

We analyzed the correlations between behavioral tests, neurotransmitter levels, and real-time PCR results. As shown in Figure 7, 18 significant correlations were observed (*p* < 0.05).

### 3.6 fMRI indicated that SNS improved the pontine ALFF change in the rat depression model

**FIGURE 8:**
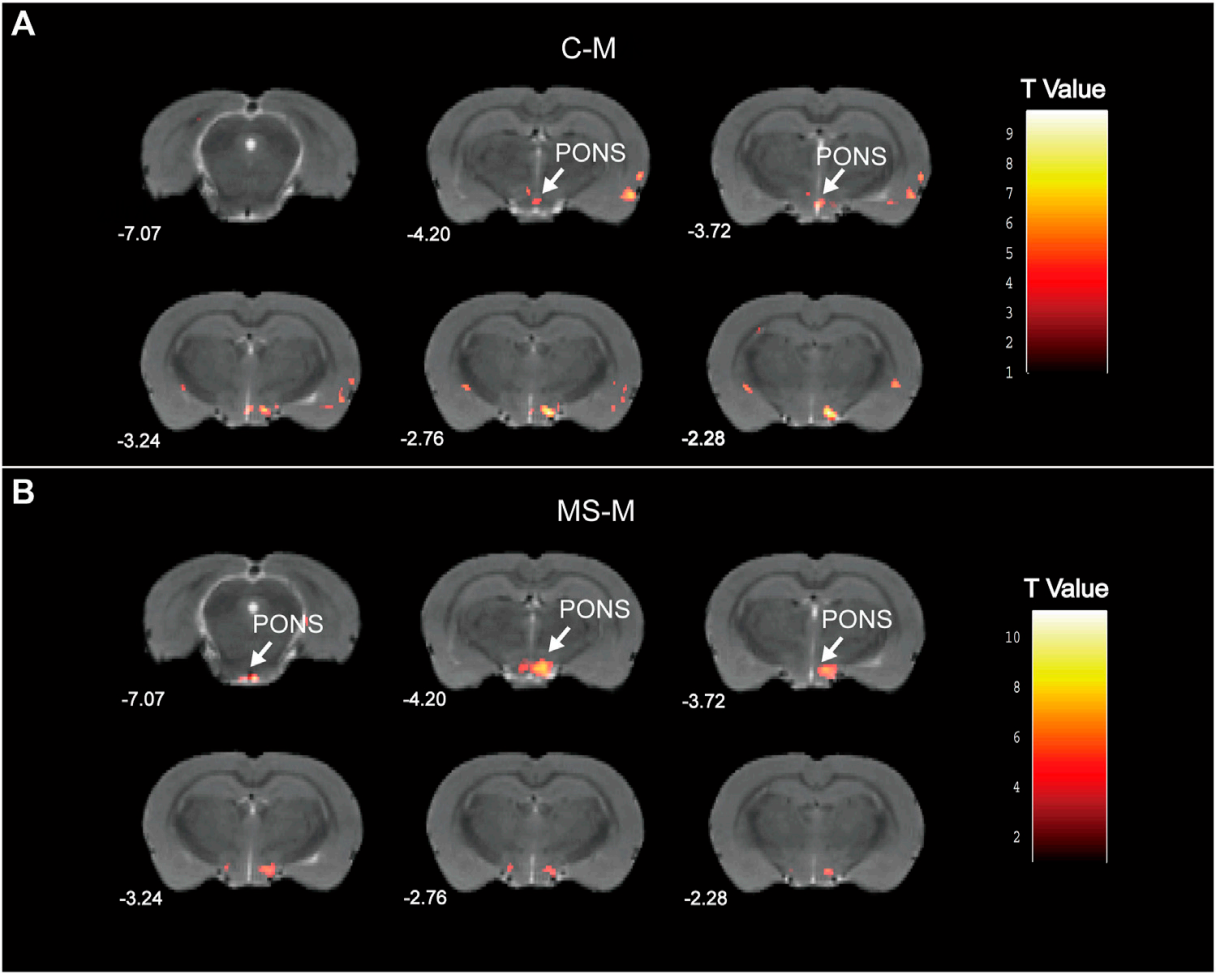
Statistical maps of voxel t values of ALFF comparisons of two chosen groups. **(A)** Decreased pontine ALFF in M group, compared to C group. **(B)** Increased pontine ALFF in MS group, compared to M group. The numbers at the bottom left of each image refer to the z coordinates in the stereotaxic space of Paxinos and Watson (2005). Color bars signify the t-value of the group analysis (brighter color indicates a larger t value). The left side of the images corresponds to the left side of the brain, and vice versa.

According to the fMRI analysis results, the pons in the M group demonstrated decreased ALFF compared to those in the C group (Figure 8A). Meanwhile, the MS group exhibited an increased ALFF in the pons compared to the M group (Figure 8B). Specific information regarding the activation points in the pons is provided in Supplementary Table S2. Hence, the SNS improved pontine ALFF changes in living rats with depression.

## 4 Discussion

Emerging evidence suggests that multiple brain regions are involved in the pathogenesis of depression. Therefore, considering the treatment characteristics of multiple organs and the targets of TCM, it is likely that the target brain regions of the antidepressant effects of SNS extend beyond the limbic system. As neuroimaging has found that the pons is related to depressive symptoms (Wong et al., 2022) and our previous study also suggested a significant correlation between learning and cognitive function in a rat model of depression (Li et al., 2018), we conducted a series of molecular biological experiments and analyses, focusing on the pons as the subject of the present study. By utilizing RNA-seq technologies and real-time PCR validation methods, we discovered that SNS has the potential to affect the expression of numerous genes in the pons, including Anxa1, Nrg1, and Psen1. By analyzing the correlation between genes, neurotransmitters, and behavior, we speculated that SNS can upregulate Anxa1 and downregulate Nrg1 and Psen1, thereby altering the release of the neurotransmitter NE, and ultimately leading to an improvement in depressive-like behavior while accompanying learning and memory impairment in the CUMS-depression model. Additionally, an fMRI experiment showed that SNS could improve pontine ALFF changes in living depressed rats. Hence, the pons is an important brain area in which SNS exerts antidepressant effects. To the best of our knowledge, this is the first study to investigate the pathology of depression and the antidepressant mechanism of SNS by targeting the pontine region. The present study suggests that SNS can not only reverse depression-like behavior induced by CUMS, but also the accompanying memory impairments. We believe that these findings collectively prove that SNS is potentially effective for treating depression.

In this study, RNA-seq technologies were used to screen for the target genes of SNS acting on the pons, resulting in a total of 49 target genes. To further understand the DEGs, GO enrichment analysis was performed. We found that these genes were closely related to functions such as regulation of biological quality (GO:0065008), somatodendritic compartment (GO:0036477), dendrite (GO:0030425), dendritic tree (GO:0097447), neuron projection (GO:0043005), neuron part (GO:0097458), mast cell granule (GO:0042629), cell projection (GO:0042995), synapse (GO:0045202), and plasma membrane bounded cell projection (GO:0120025). Although there are currently no experimental studies on the antidepressant targets of SNS in the pontine nucleus, many scholars have used network pharmacology methods to explore antidepressant targets by searching different databases. The predicted antidepressant targets of SNS based on network pharmacology (Zong et al., 2019; Zhu et al., 2024) were also related to the function of plasma membrane-bound cell projections, neuron projections, and dendrites, which is consistent with the results of our study. Because our results were based on experimental validation, our findings will enrich the content of the SymMap database (Wu et al., 2019) and ETCM (Xu et al., 2019).

The present study showed that multiple pontine genes, including Anxa1, Serpinf1, Arrb1, Cplx2, Nrg1, and Psen1 were altered in a model of depression. Annexin A1, encoded by Anxa1, is an endogenous ligand of formyl peptide receptor (FPR) 2/3, a member of the phospholipid and calcium-binding protein family. It plays a definite role in the delayed early inhibitory feedback of glucocorticoids (GC) within the pituitary gland and is associated with behavioral disorders such as anxiety. Anxa1 gene knockout mice showed histological changes and reduced neuronal death in hippocampal slices (Peritore et al., 2020). This study showed expression of Anxa1 in the pons, which was inconsistent with the functional attributes of Anxa1. However, upregulated Anxa1 expression has been reported in the hippocampal region when depression occurs, and the hippocampus belongs to the limbic system. To date, there have been no studies on the expression of the Anxa1 gene in the pons of a depression model. Therefore, we speculate that there is a possibility that the expression of Anxa1 in the pons and hippocampus is inconsistent when depression occurs. Serpinf1 encodes the pigment epithelium-derived factor (PEDF), a multifunctional glycoprotein with antidepressant effects. PEDF overexpression significantly upregulates 5-hydroxyindoleacetic acid and 5-hydroxytryptamine levels in mice (Bai et al., 2022). The inhibition of Serpinf1 expression can lead to depressive behavior (Jiang et al., 2021). This study showed that SNS improved the downregulation of Serpinf1 expression in a rat model of depression, which may be an antidepressant mechanism of SNS. β-arrestin1 encoded by the Arrb1 gene is a potential biomarker for the response to antidepressant therapy in depression. Arrb1 mutation may affect the response of patients with depression to antidepressant therapy (Chappell et al., 2022). Crisafulli et al. investigated 10 nucleotide polymorphisms in 36 genes associated with major depressive disorder and bipolar disorder as well as their response to treatment. Cplx2 is one such gene (Crisafulli et al., 2013). Nrg1 performs a diverse array of functions, including the regulation of neural development and neurotransmission within the nervous system (Ding et al., 2023). Nrg1 is a potential biomarker for various clinical subtypes of depression and bipolar disorder (Levchenko et al., 2020). It can also expedite the forgetting of fear memories and facilitate the induction of long-term depression in adult mice (Cao et al., 2021). Psen1 is a Presenilin gene, and its mutation is associated with both early and late onset of Alzheimer’s disease (AD) (Tortelli et al., 2021), which has been found to be highly correlated with depression (Wang et al., 2020; Martin-Sanchez et al., 2021). PSEN1-L226F mutation is closely related to the occurrence of mental disorders and subsequent cognitive impairment, as well as the occurrence of depressive symptoms (Bartesaghi et al., 2020). This study showed that SNS could downregulate the expression of Nrg1 and Psen1, indicating an improvement in depression, which is consistent with the results of previous studies. Numerous studies have reported that the neurotransmitters 5-HT and NE play a significant role in the onset and progression of depression, and that the Anxa1, Serpinf1, Arrb1, Cplx2, Nrg1, and Psen1 genes regulate the levels of the neurotransmitters 5-HT and NE, thereby affecting behavioral indicators in rats and mice (Szapacs et al., 2004; Glynn et al., 2010; Levchenko et al., 2020; Peritore et al., 2020; Martin-Sanchez et al., 2021; Bai et al., 2022; Chen et al., 2022). In this study, we conducted a correlation analysis between six genes, neurotransmitters, and behavioral indicators, which referred to the method of correlation analysis in the SympGAN (Lu et al., 2023). We found that Arrb1, Nrg1, Anxa1, Psen1, and Serpinf1 had a statistically significant correlation with neurotransmitter levels and behavioral indicators. Since SNS had no significant effect on Arrb1, 5-HT, and DA, it may improve the level of pontine NE by regulating the genes Anxa1, Nrg1, and Psen1, thereby exerting antidepressant effects and improving cognitive function.

Although our study suggests that SNS exerts antidepressant effects by improving the expression of the three genes and NE in the pons, the specific mechanisms involved are not yet fully understood. Little research has been conducted on the impact of knocking out these six genes on 5-HT and NE levels as well as on behavior, which hinders the ability to effectively establish the role of these genes in the onset and progression of depression and their response to drug treatment. However, further comprehensive studies are required to confirm these findings. In the OFT, the model group exhibited increased rearing and total distance moved, which appears to contradict the conventional behavioral manifestations of depression. However, we believe that this was due to the accompanying anxiety state exhibited by some depressed rats, consistent with the conclusion that anxiety symptoms may occur during depression. Upregulation of Anxa1 in some rat models of depression may be explained by its association with anxiety.

## 5 Conclusion

In summary, this study indicates that SNS effectively reverses depression-like behavior and memory impairment in rats by regulating gene expression in the pons. SNS is a widely used formula in traditional Chinese medicine for the treatment of depression, with minimal side effects and high safety. Therefore, its promotion and adoption for the treatment of depression worldwide is anticipated. The active component monomers of SNS have the potential to offer new insights into the development of novel drugs against depression.

## Data Availability

The data presented in the study are deposited in the NCBI repository, BioProject accession number PRJNA1074724.
