# Supplementary material for: High throughput mRNA sequencing reveals potential therapeutic targets of Si-Ni-San in the pons for a stress-induced depression model
Source: Front Pharmacol. 2024 Jul 29;15:1383624. doi: 10.3389/fphar.2024.1383624 (PMC11317778; doi:10.3389/fphar.2024.1383624)
Supplement: Supplementary file 1 [file Table1.DOCX]

Supplementary Material

High Throughput mRNA Sequencing Reveals Potential Therapeutic Targets of Si-Ni-San in the Pons in a Stress-induced Depression Model

Li Junling*, Zhang Yan, Li Te, Nie binbin, Qi Fang, Chen Qijun, Chen Tianxing, Liu Yuhang, Li Gaifen, Li Yubo*

*** Correspondence:** Li Yubo:liyubo123456@126.com

Supplementary Table 1 The 14 gene nodes with degree in the function-gene network

| Gene | Related GO term | degree | M_vs_C(log2FoldChange) | MS_vs_M(log2FoldChange) |
| --- | --- | --- | --- | --- |
| Cplx2 | regulation of biological quality, somatodendritic compartment,dendrite,dendritic tree,neuron projection,neuron part,mast cell granule,cell projection,synapse,plasma membrane bounded cell projection | 10 | 1.695512832 | -1.640361223 |
| Serpinf1 | regulation of biological quality, somatodendritic compartment, neuron projection, neuron part,cell projection, plasma membrane bounded cell projection | 6 | -1.937977429 | 1.069398808 |
| Psen1 | regulation of biological quality, somatodendritic compartment, dendrite,dendritic tree,neuron projection,neuron part,cell projection,synapse,plasma membrane bounded cell projection | 9 | 1.0009437 | -1.222244484 |
| Sarm1 | somatodendritic compartment,dendrite,dendritic tree,neuron projection,neuron part,cell projection,synapse,plasma membrane bounded cell projection | 8 | 1.382582136 | -1.122671979 |
| Nrg1 | regulation of biological quality, somatodendritic compartment, dendrite,dendritic tree,neuron projection,neuron part,cell projection,synapse,plasma membrane bounded cell projection | 9 | 1.123822955 | -1.180860648 |
| Rims4 | regulation of biological quality, synapse | 2 | 1.989692637 | -1.928234759 |
| S100a4 | neuron projection,neuron part,cell projection,plasma membrane bounded cell projection | 4 | -1.218101587 | 1.111829179 |
| Kcnip3 | somatodendritic compartment,dendrite,dendritic tree,neuron projection,neuron part, cell projection,synapse,plasma membrane bounded cell projection | 8 | 1.376235469 | -1.303709497 |
| Anxa1 | regulation of biological quality,mast cell granule,cell projection,plasma membrane bounded cell projection | 4 | -2.086932383 | 1.134405391 |
| Vim | neuron projection,neuron part, cell projection, plasma membrane bounded cell projection | 4 | -1.745216127 | 1.021611683 |
| Shank1 | regulation of biological quality, somatodendritic compartment,dendrite,dendritic tree, neuron projection,neuron part,cell projection,synapse,plasma membrane bounded cell projection | 9 | 1.289950999 | -1.642881734 |
| Arrb1 | regulation of biological quality, somatodendritic compartment, dendrite, dendritic tree, neuron projection, neuron part,cell projection,synapse, plasma membrane bounded cell projection | 9 | 1.085897419 | -1.442008152 |
| Sptbn2 | regulation of biological quality, somatodendritic compartment,neuron part | 3 | 1.038038525 | -1.028947797 |
| Trim9 | regulation of biological quality, somatodendritic compartment,dendrite,dendritic tree, neuron projection,neuron part, cell projection, synapse,plasma membrane bounded cell projection | 9 | 1.135166279 | -1.327187043 |

Supplementary Table 2 The specific information of the activation point in pons

|  | **ROI** | **Ke** | **MAX_T** | **X** | **Y** | **Z** |
| --- | --- | --- | --- | --- | --- | --- |
| **C-M** | Pons | 89 | 4.938 | -0.2284 | 8.1292 | -3.7179 |
| **MS-M** | Pons | 28 | 5.7594 | 0.0773 | 9.1681 | -7.0779 |
|  | Pons | 21 | 5.8707 | 0.0773 | 9.1681 | -7.0779 |
|  | Pons | 5 | 4.4939 | 0.0839 | 8.9098 | -7.5579 |
|  | Pons | 4 | 4.79 | 0.0839 | 8.9098 | -7.5579 |
|  | Pons | 1 | 4.8626 | -0.2218 | 8.0125 | -4.1979 |
